# Type and Proximity of Green Spaces Are Important for Preventing Cardiovascular Morbidity and Diabetes—A Cross-Sectional Study for Quebec, Canada

**DOI:** 10.3390/ijerph13040423

**Published:** 2016-04-14

**Authors:** Roland Ngom, Pierre Gosselin, Claudia Blais, Louis Rochette

**Affiliations:** 1 Geoimpacts Consulting, 111 Rue de la Chasse Galerie, Québec, QC G1B 1Y2, Canada; 2 Institut National de la Santé Publique du Québec, 945, Avenue Wolfe, QC G1V 5B3, Canada; pierre.gosselin@inspq.qc.ca (P.G.); claudia.blais@inspq.qc.ca (C.B.); louis.rochette@inspq.qc.ca (L.R.); 3 Institut National de la Recherche Scientifique, 490, Rue de la Couronne, Québec, QC G1K 9A9, Canada; 4 Faculty of Medicine, Université Laval, 1050 Avenue de la Médécine, Québec, QC G1V 0A6, Canada

**Keywords:** green spaces, cardiovascular diseases, diabetes, typology, primary prevention

## Abstract

This study aimed at determining the role of proximity to specific types of green spaces (GSes) as well as their spatial location in the relationship with the most morbid cardiovascular diseases (CVD) and diabetes. We measured the accessibility to various types of GS and used a cross-sectional approach at census Dissemination Area (DA) levels in the Montreal and Quebec City metropolitan zones for the period 2006–2011. Poisson and negative binomial regression models were fitted to quantify the relationship between distances to specific types of GS and CVD morbidity as well as some risk factors (diabetes and hypertension) while controlling for several social and environmental confounders. GSes that have sports facilities showed a significant relationship to cerebrovascular diseases: the most distant population had an 11% higher prevalence rate ratio (PRR) compared to the nearest, as well as higher diabetes risk (PRR 9%) than the nearest. However, the overall model performance and the understanding of the role of GSes with sport facilities may be substantially achieved with lifestyle factors. Significantly higher prevalence of diabetes and cerebrovascular diseases as well as lower access to GSes equipped with sports facilities were found in suburban areas. GSes can advantageously be used to prevent some CVDs and their risk factors, but there may be a need to reconsider their types and location.

## 1. Introduction

The global burden of disease has dramatically shifted from communicable, maternal, perinatal, and nutritional causes to non-communicable diseases (NCDs) [1]. Cardiovascular diseases (CVD) account for the most NCD deaths worldwide, taking the lives of 17.5 million people annually. After cancers and respiratory diseases, diabetes follows as highest contributor to NCD deaths with 1.5 million [1].

There is an urgent need to develop strategies that will help reduce this burden. Implementing primary prevention strategies using environmental resources may be a sustainable option for this purpose. What could be the role of green space (GS) in the implementation of such strategies? Before investigating this question, it may be useful to define a green space. This question is almost philosophical given the variety of existing definitions. GSes are usually classified, as in the Oxford dictionary, as an open area with the particularity that it partly or completely contains vegetation. It is usually seen as a discontinuity of the urban or built-up areas. The typology of GSes vary as they fulfill many ecological and social services. Among various well-known types of GS, we can cite parks (urban parks, formal gardens and country parks), natural and semi-natural GS (woodlands, urban, forestry, scrub, grasslands, wetlands), green corridors (towpaths along canals and riverbanks, cycle ways and disused railway lines), amenity green space (includes informal recreation spaces), and outdoor sports (natural or artificial surfaces used for sports and recreation. Green spaces also include outdoor sports pitches, tennis courts and bowls, golf courses, athletics fields, playing fields, allotments, community gardens, and even churchyards. We can easily understand that each of those types of GS has a specific design and its surface area varies accordingly.

It is suggested that the likelihood of using public open spaces increases with their proximity and their specific type in terms of the features they offer and/or how they are designed to optimize their utilization by the population [2]. Yet these characteristics are usually ignored or incompletely analyzed in studies that examine the relationship between green spaces and physical health, particularly CVDs. Most of the studies are restricted to a metric measurement of GS accessibility, and approaches to measure this accessibility vary considerably. Moreover, while many studies have been conducted to better understand the relationship between GS and mental health, including systematic reviews [3,4,5,6], few have been developed to establish the relationship between CVD and GS [7,8,9].

Air pollution and extreme heat are recognized CVD risk factors [10,11,12,13,14], and GSes are known to reduce air pollution and moderate urban heat [15]. However, there is still a lack of knowledge of how this benefits CVD and its risks factors. GSes are also known to promote physical activities [16], which is one of the most important factors helping to reduce CVD morbidity and mortality [17]. There is also a gap in understanding which type of GS is related to the reduction of CVD, diabetes and hypertension risks, since this may be associated with the intensity of physical activity that is possible to realize within a specific type of GS. Finally, the multiplicity of roles attributable to GSes and their relations to each CVD may be confusing. The ecological functions of GSes may be highly related to their size (surface area), the vegetation density and type of space, while the social functions of GSes and their ability to attract people may additionally depend on the equipment allocated to a GS.

The aim of this study was to develop a conceptual framework of GS attractiveness based on GS typology and proximity and to analyze the relationship with CVDs and their risks factors. Under the assumption that GS types and accessibility vary with possible consequences on their relationship with CVD and their risks factors, focusing on the resulting spatial disparities was also an important objective. For this reason, the study was conducted in two census metropolitan areas (CMA). A CMA is formed by one or more adjacent municipalities centered on a large urban area (known as the urban core). The CMA of Montreal is the main catchment area of the province of Quebec (Canada) and is the most important economic center. The CMA of Quebec City is the second biggest in the province. The choice of these cities was also motivated by the fact that this study was nested within a larger research initiative on adaptation to climate change and health in the province of Quebec.

## 2. Methods and Data

### 2.1. Health Data

In a cross-sectional approach, we used a sample of data from the Quebec Integrated Chronic Disease Surveillance System (QICDSS) of the Institut national de santé publique du Québec, which is a linkage of five health administrative studies previously described [18], to define selected health variables for individuals aged 20 years and older living in the CMAs of Montreal and Quebec City from 2006 to 2011. The exposure period to GS was also from 2006 to 2011. There were a total of 3,920,000 individuals involved in the two study sites.

We used validated case definitions of coronary heart disease, cerebrovascular disease and heart failure and their risk factors (hypertension and diabetes) to define the prevalence of these CVDs [19,20,21,22,23]. These case definitions mainly use diagnostic codes from the International Classification of Diseases (ICD-9 and ICD-10-CA). Diabetes (types 1 and 2) and hypertension were used as explanatory variables for CVD.

Individual data on diseases were summarized at the Dissemination Area (DA) level. Administrative space boundaries, or those from censuses such as DAs, may be less accurate than individual-based information. However, they may be convenient in the case of privacy concerns, as it is with the health data used for this study, specifically for geographic representations. The DA is a basic Canadian census unit encompassing on average between 400 and 700 individuals [24]. It offers stronger stability in terms of population size than the census bloc, which is the smallest census unit in Canada. They are the smallest standard geographic area for which all census data are disseminated, and provide a higher socioeconomic homogeneity than the smaller census units [24,25].

### 2.2. Green Space Data

**Figure 1:**
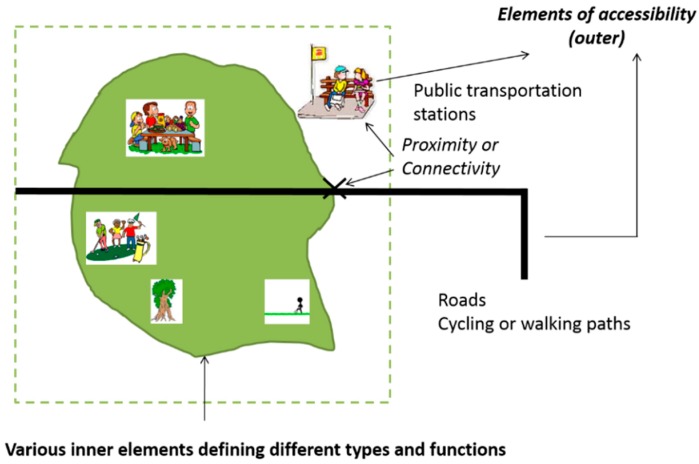
A conceptual framework for linking green space typology and accessibility.

Physical representation of a GS is usually made through a polygon representing an opened area covered with vegetation (being ligneous or not). Aside from type of vegetation which can be more or less attractive, we considered that additional elements inside the polygon may play an important role in attracting users. We believe that outer elements in proximity or physically connected to the vegetation polygon also intervene in the definition of the accessibility of a GS in complement to the metric distance between individuals’ homes (postal codes) and GS. We hypothesized that all those outer and inner elements contribute to the overall likelihood of a GS to be used by the population (Figure 1).

In order to illustrate specific types of GSes in the database used for analysis, data from the Desktop Managing Technologies Inc. (DMTI) for 2011 were used as the main source to build the GS variable. We used the CanMap product suite which is the richest, most detailed mapping content available for Canada. Companies like Garmin and Google Allstream depend upon DMTI data [26]. This implies that the spatial resolution of the data that were used in this study is exactly the same that one can visualize in Garmin or Google products. The CanMap Postal Code Suite is the most complete postal geography available in the market. The Six Digit Postal Code File which is a precision-based point file representation of postal codes across Canada, has a scale of 1:50,000. The CanMap Parks and Recreation produced by DMTI, represents over 1600 national, provincial and territorial parks and over 14,000 recreational areas across Canada. Includes: national, provincial, territorial, ecological reserves, wilderness parks and areas, wildland parks, grizzly bear sanctuaries, recreational areas, municipal parks, private parks and golf courses, protected areas, heritage parks, natural parks, park reserve boundaries and points. CanMap Route Logistics, based on the highest quality street map data available, provides an accurate map fabric that includes among others road directions, type, transportation route restrictions layer, travel time, and travel speed estimates based on road elevations. The Enhanced Points of Interest (EPOI) file is a national database of over 1 million Canadian business and recreational points of interest. Engineered using CanMap^®^ Streetfiles, each EPOI has been accurately geocoded and precisely placed. Nationwide features available include coordinate location (*x, y*).

The presence of vegetation within open areas was monitored using spatial queries under ArcGIS of the Environmental Systems Research Institute (ESRI). Spatial queries were also used to illustrate built-up elements inside the polygons. These included natural functions as represented by regional/national parks and wooded lands; hedonic functions identified through parks with sports facilities; and those dedicated to fairs and other fun activities. Some of the GS elements found both inside and/or outside the polygons and also related with the metric accessibility to GSes were walking and cycling paths as well as all types of roads crossing a GS or polygon boundary. Backyards were not included in this study. The surface area of each polygon representing GS, despite not specifically representing a type of GS, was considered as a distinct function of GS. The minimum surface of the GS was 2.5 m^2^ and the maximum was 720 km^2^ with a mean value of 0.77 km^2^.

A Euclidian method was used for the calculation of the nearest distance (maximum of 200 m) from each GS boundary to the nearest public transportation stations (bus and/or metro). The final calculation of the distance between the centroids of the six-digits postal codes and the nearest GS boundary (not centroid) integrated weighting factors such as topography, artificial restrictions such as highways and natural restrictions posed by rivers. Road restrictions were included in the DMTI database. The Network Analyst module of ArcGIS was used to calculate the weighted distances from individuals’ postal code location centroids (point of departure) to the nearest GS (destinations). The weighting was defined by the topography, as well as road directions and restrictions described within the DMTI database.

As a summary of these analyses and in relationship with the conceptual framework that was developed, the nearest distance to the following types of GS were considered (a type may not be exclusive):

- A GS with natural functions (provincial or national parks and woodlands).
- A GS with sport facilities (golf courses or any sport facilities).
- A GS used for fairs and other fun activities (any type of park, excluding provincial and national parks as well woodlands and GSes with sport facilities).
- A GS crossed by walking or cycling tracks (any type accessed and completely or partly crossed by walking or cycling tracks).
- A GS crossed by roads (any type accessed and completely or partly crossed by a road)
- A GS accessible by public transportation (bus or metro).
- A GS surface area illustrating its size.
- An additional variable considering the distance to any type of GS (regardless of its specificity, but only considering the presence of vegetation) was also analyzed.

**Figure 2:**
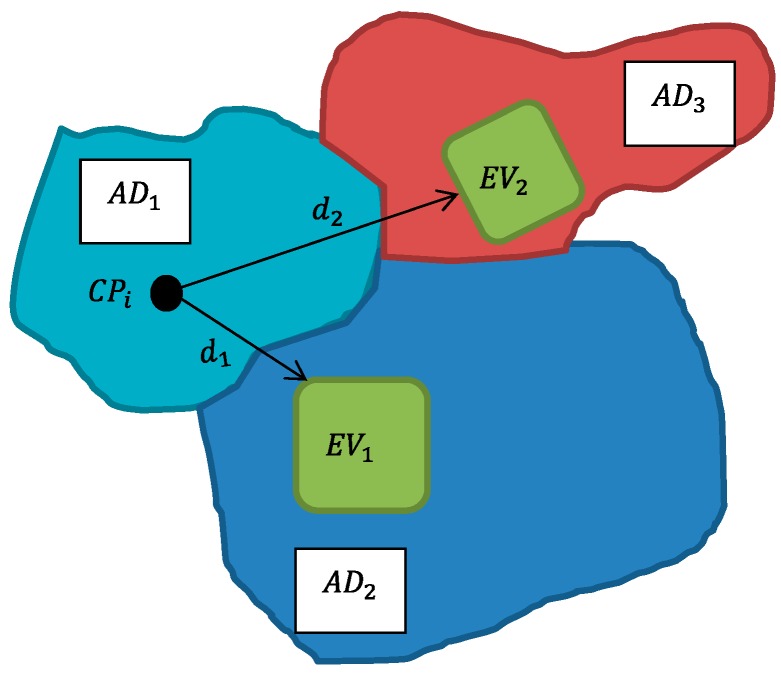
Conceptual framework of distance matrix to green spaces in Dissemination Areas. *AD* = Dissemination Area; *EV* = Green Space; *CP* is a six-digit postal code; d_1_ and d_2_ are weighted distances from a postal code to the boundary of a green space.

In Figure 2, the individual *i* whose location is the postal code *CP_i_*, might not have a GS in its DA, which justifies the relevance to consider GS *EV*_1_ nearby (being shorter), but within another DA (for the closest). An inference of GS accessibility (DA’s mean distance to the nearest GS) was obtained using this method. The distances to GS (according to their specific types as defined earlier) were transformed into quartiles for 7550 DAs.

### 2.3. Social and Demographic Predictors of Cardiovascular Diseases

Social and material deprivation scores, developed at the Institut national de santé publique du Québec and initially calculated at the DA level were considered as a proxy of the socioeconomic status (SES) [25,27]. The material deprivation score integrates income, education and employment variables from the national Canadian census of 2006.

In the province of Quebec, immigrants may be at lower risk of CVD. This so-called immigration bias or healthy immigrant effect may be an important variable in a country such as Canada where some urban areas incorporate large proportions of immigrants [28,29]. In order to illustrate a potential immigration bias, we have considered the ratio of recent immigrants in the DAs, *i.e.*, within the country in the last 10 years, as per the 2011 national census.

Total population counts at the DA census division level were obtained from Statistics Canada [24] and population densities were calculated. Population density is a proxy for the level of urbanity of an area. The most urbanized DAs are the smallest in terms of area, and therefore the densest. Services provided within denser areas may differ from those offered in less dense settings. Those services may include specific types of GS. Moreover, population densities are indirectly related to pollution [30,31].

### 2.4. Environmental Predictors of Cardiovascular Diseases

We used three variables to characterize exposure to ambient air pollution. First, as some ultra-fine particles are emitted directly from industries, data from the National Pollutant Release Inventory (NPRI), produced by Environment Canada for the year 2011 [32], was examined to measure the exposure to major industrial sources of pollution; Euclidean distances were calculated for each postal codes in the QICDSS. Mean distance values to the sources were then calculated for each DA. Second, it has been shown that levels of ultra-fine particles are similar to background levels at distances greater than 300 m from major roads [33,34]. In order to consider pollution due to road traffic, data on the major roads and highways were used and their Euclidean distances to postal codes calculated. Mean distance values for each DA were considered as the last data format for analysis. Finally, average values data for the amounts of PM_2.5_ particles were calculated for the study period 2006 to 2011. A mean value of PM_2.5_ emission was calculated at the DA level.

Domestic air conditioning has been proved to be associated with a lower cardiovascular morbidity [35]. Data estimating the presence of air conditioning in households were provided by Hydro-Québec. This estimate is based on a comparison of the variation and pattern of the power consumption during summer.

Urban heat has been identified as having a physiological relationship with the mortality of CVD [36], notably in Montreal [37]. Urban heat island data were obtained through the modeling of remote sensing data for the years 2010–2011. A mean value of urban heat was calculated for each DA [38].

The relationship between proximity to indoor sports centers, such as fitness centers, and CVD has rarely been studied. However, under the assumption that this variable is concurrent with the proximity to GS, it has been added as a confounding variable in this study. A list of indoor sports centers, mainly composed of private fitness centers, was obtained from the Quebec consumers’ protection bureau. Six-digit postal codes in this list helped calculate weighted distances between these indoor sports centers and the postal codes of individuals incorporated within the QICDSS. Mean weighted distance of individuals to indoor sports centers were calculated for each DA.

Finally, the role of GS in the mitigation of air pollutants, while often of importance, seems to be variable across several settings, depending on the tree species, meteorology and climate; little is known on this topic in temperature climates [39,40].

### 2.5. Analysis

The selected CVD were considered as outcome (cerebrovascular diseases, heart failure, ischemic heart disease), in addition to morbidity and mortality variables that were regrouping all the selected CVDs. Diabetes and hypertension cases were also used as outcomes. Cases were aggregated at the DA level and statistical analyses were made using the DA as the observational unit.

Specific distinct regression Poisson or negative binomial models were fitted for each specific function or type of GS described in Section 2.2. For each of those models, GSes were included as a predictive variable. Negative binomial regression models were preferred in case of over-dispersion. Prior to Poisson analysis, Cochran Mantel Haenszel tests were performed to illustrate the effect of gender on outcomes. Following the results of those analyses, stratification by age was applied for CVD and hypertension, and age and gender stratification was applied for diabetes. Those stratifications were applied under the form of the population size of each age group/and gender for each DA (expected cases) and integrated as an offset within the models.

Social and environmental predictors described earlier were included as such in the models, except for PM_2.5_ particles, air conditioning use and urban heat islands. Because of their assumed modifier effect, they were used for interactions between both the proximity to a GS without specific function or type, and to a GS with a specific typology. These models were subsequently divided into separate multivariate models that included all the other relevant predictors.

Bivariate Moran’s I analysis is a type of spatial analysis that produces a map of local indicator of spatial association (LISA) or clusters between two variables. Local clusters in the resulting map mean that there are areas that have higher or lower values than is to be expected by chance alone [41]. We wanted to obtain spatial clusters of high distances to GS and high cardiovascular prevalence rates on one side, and low distances to GS and low cardiovascular prevalence rates on the other side. As input, we integrated two variables:

- Empirical Bayesian standardized morbidity rates (SMR) maps of the statistically relevant CVD or their risk factors. The relevance was based on the significance of the relationship between GS and the CVD or their risk factors within the Poisson or negative binomial regression models. Bayesian analysis helps to better control the variance due to the discrepancies between total population sizes of the different DAs [42].
- The second input was the GS variables that were relevant in Poisson or negative binomial regressions models.

## 3. Results and Discussion

### 3.1. Results

Prevalence of ischemic heart diseases and diabetes was higher than that of heart failures and cerebrovascular diseases (Table 1). The highest age standardized and crude rates were those of ischemic heart diseases in Quebec with 8.31% (99% CI: 8.22–8.40) and 10.58% (99% CI: 10.46–10.69), respectively (Table 1), whereas diabetes showed the highest age standardized and crude rates in Montreal with 8.26% (99% CI: 8.20–8.31) and 9.10% (99% CI: 9.04–9.17), respectively.

GS without the definition of a specific type showed no significant correlation with CVD. None of the interactions between distance to GS (without specific function or type) and the following environmental variables showed a significant correlation with CVD: urban heat, level of PM_2.5_ particles, and air conditioning.

Among the various GS types, only GSes with sport facilities showed a significant relationship with diabetes and cerebrovascular disease morbidity (Table 2 and Table 3). For cerebrovascular diseases, the most distance to this type of GS showed a prevalence rate that was 11% (95% confidence intervals 1.01–1.22) higher compared to the least distant (Table 1). For diabetes, this ratio was 9% (95% confidence intervals 1.03–1.13) higher for the most distant to those GS equipped with sports infrastructure, compared to the least distant (Table 3). The other CVDs (heart failure and ischemic heart disease) and hypertension were not significantly associated with GS, regardless of type.

**Figure 3:**
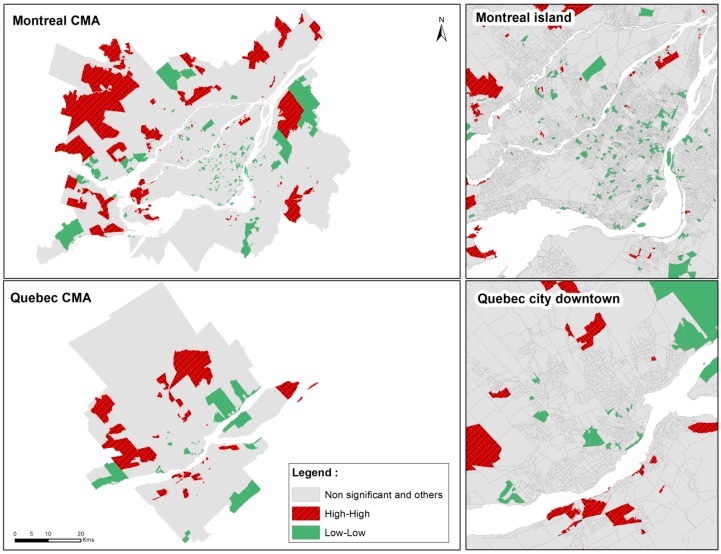
Bivariate local Moran maps of cerebrovascular standardized morbidity rates and distance to green spaces with sport facilities for adults ≥20 years living in the CMAs of Montreal and Quebec City, 2006−2011. CMA: Central Metropolitan Area; SMR: standardized morbidity rates; GSes: green spaces; High-High: Cluster of highest SMR and highest distance to GSes with sport facilities; Low-Low: Cluster of lowest SMR and lowest distance to GSes with sport facilities.

**Figure 4:**
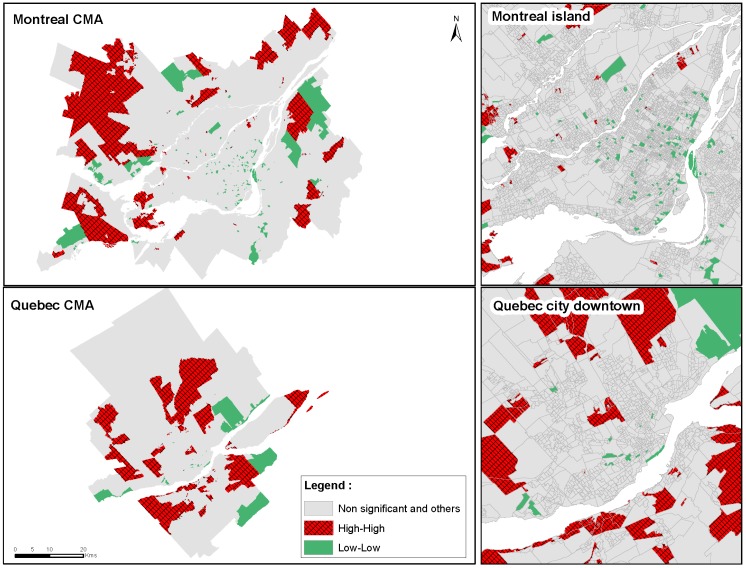
Bivariate local Moran maps of diabetes standardized morbidity rates and distance to green spaces equipped with sport facilities for adults ≥20 years living in the CMAs of Montreal and Quebec City, 2006−2011. CMA: Central Metropolitan Area; SMR: standardized morbidity rates; GSes: green spaces; High-High: Cluster of highest SMR and highest distance to GSes with sport facilities; Low-Low: Cluster of lowest SMR and lowest distance to GSes with sport facilities.

The bivariate local Moran maps showed a higher number of hot spots (high-high clusters or higher distance to GS and higher level of diseases) in peripheral and less dense areas for cerebrovascular morbidity and diabetes cases (Figure 3 and Figure 4). It suggests that those areas have a higher distance to GSes equipped with sport infrastructure and a higher prevalence of cerebrovascular diseases (Figure 3) or diabetes (Figure 4), whereas the areas with lower prevalence of cerebrovascular diseases and diabetes are significantly associated with a lower distance to GSes that have sports facilities. This structure is confirmed by the multivariate regression models where the densest DAs showed significantly lower PRRs than the least dense DAs (Table 1 and Table 2).

### 3.2. Types of Green Spaces that Promote Vigorous Physical Activities May Be Important to Prevent Cardiovascular Diseases and Diabetes

Only proximity to GSes that can stimulate vigorous physical activities have been shown, in this study, to play a role in reducing cerebrovascular diseases as well as an important CVD risk factor, namely diabetes. This may underline the importance of this type of GS at the community level. Lifestyle factors may actually achieve the overall contribution of explanatory variables of the model. They would probably complement the understanding of the role of GS types and proximity. Despite the absence of lifestyle covariates such as smoking, eating habits and even level of physical activity in the regression models, these results are in line with many studies that showed the importance of GS in stimulating vigorous physical activities.

One of the important elements of this study supporting the key role of GSes harbouring sport facilities is the less significant role of indoor facilities (sport facilities) in the regression models. Interestingly, Cohen *et al.* conducted a study in Los Angeles, identifying parks as the most common place where people exercised [43]. Kaczynski and Henderson demonstrated that outdoor settings more frequently have positive and significant associations with higher levels of or incidence of physical activities than primarily indoor settings [44].

A hypothesis that has not been explored in this study, but that is worth mentioning is that GS may indirectly contribute to solving CVD and diabetes problems through improving mental health. It is agreed that mental illness and psychosocial stress are risk factors for CVD [45]. Some findings indeed suggest that physical exercise in pleasant environments (such as green environments) may have a greater effect than exercise alone on blood pressure, an important measure of cardiovascular health as well as on mental health [3]. This assumption brings back the notion of park esthetic amenities combined with park facilities. Green spaces with sport facilities may be highly attractive as recreational sites, and can complement the other built-up features that stimulate physical activities for better health more efficiently. More and more evidence of GS effects on promoting positive mood [46], reducing mental stress [47,48], avoiding anxiety caused by urban noise [49], and enhancing social support [50] are being provided.

Another element in our study that supports the role of GSes with sport facilities is the absence of significant associations between CVD and other risk factors with those GSes without a specific type. The number of such GSes in the database is obviously higher. Actually, GSes harbouring sport facilities only represent 0.95% of the total GS features of the study, the biggest part being represented by GSes with a natural function. Kaczynski and Henderson also demonstrated that parks with more features were more likely to be used for physical activity and that park facilities were more important than park amenities for physical activities [51]. This last statement may help in understanding the spatial difference between suburbs and downtowns of the study sites as related to the cerebrovascular diseases and diabetes.

Other studies have shown the importance of the environment of suburban populations in reducing their physical activities compared to people living downtown [52,53,54]. In general, downtowns offer higher possibilities for various types of physical activities including intensive and moderate ones such as walking [4,55]. This presumed higher possibility of moderate activities may be playing a confounding or residual role favoring better health in the most urbanized areas [4]. One can legitimately question the results of our study in regards to other attractive urban elements (e.g., shopping streets, pedestrian streets) not included within this study, and that can promote a higher level of walkability in the most urbanized areas, thus creating confusion with the role of GSes. The role of the type of GS in the suburban population’s cardiovascular health is not yet clearly defined. Despite a higher proximity to GSes, mainly to woodlands with higher surface areas, the worse state of cardiovascular health of the suburban population may be associated with the lack of attractive (*i.e.*, features inside or connected to the vegetation) and easily accessible GSes, particularly those that stimulate vigorous physical activity as suggested by the results of this study.

### 3.3. Are Green Spaces’ Ecological Services Less Important than Social Services for Preventing Cardiovascular Diseases?

Bigger surface areas of GSes are an indicator of the potential of GS to reduce pollution and urban heat particularly in less dense settings where woodlands are more present than in most urbanized settings [56,57]. Given the complexity of ecological processes between the atmosphere and vegetation, it may be presumptuous to conclude that there is no effect of GS vegetation on CVD. Some of the elements such as the type of vegetation and the wind direction may render the measurement of the relationship between GS and ambient air pollution more difficult to assess. The results of this study suggest that the social role of GSes, particularly those with adequate facilities for vigorous physical exercise, may be more important to help prevent CVD than the simple presence of vegetation. Moreover, the presence of massive woodlands with no or limited facilities may reduce the population’s ability to use a GS for physical activities. This may be more impactful and challenging in less urbanized settings.

### 3.4. Implication for Public Health

Measuring access to and use of GSes for public health purposes is complex given the various services provided by GS, including their ecological and social services. Measurement issues of GS accessibility and attractiveness (*i.e.*, the reasons why people go to a specific type of GS) vary, and this probably impacts the measurement of their usefulness for health purposes, particularly for CVD and their risks factors. At the global and domestic levels (municipalities), the accessibility of GS is commonly reduced to the measurement of green surface per capita [58,59], which may not reflect the importance of distinguishing the type of GS in terms of its accessibility.

This study has demonstrated that the option to consider the typology of GS as a function of what it contains in terms of physical features and their accessibility may be more convenient as a measurement reference to help implement adequate GS structures for the primary prevention of CVD and diabetes. Absence or low presence of certain types of GS in less urbanized settings indicates a spatial disparity that may put to question the choice of maintaining certain types of GSes in specific settings. More than ever, there is a need for collaboration between city planners and public health authorities to promote sustainable environments that can effectively benefit the population’s cardiovascular health.

### 3.5. Limitations of this Study

The main limitation of this study is the absence of factoring in lifestyle, as well as actual data on the usage of GS. Lifestyle factors likely contribute more to diabetes and CVD than the simple presence or proximity of GS. This implies that the overall performance of the explanatory variables would probably be higher if lifestyle factors were integrated in the models. Even if the case definitions of each CVD studied here are validated, each one contains some limitations such as low sensitivity, especially for cerebrovascular disease (68%) [18]. Data and analysis of walkability, as associated with urban features, could also render the results more robust. GSes with more open lawn areas (or other open space) may accommodate diverse physical activities, which may contribute to a lower prevalence of CVD; unfortunately, we did not have any data specifying the presence of open lawn areas. However, among the types of green spaces defined, we suspect that green space used for fairs and other fun activities, as well as golf courses, can be considered an open lawn space that accommodates diverse physical activities. The measurement of pollution levels is a complex issue and the association with GS may call for more sophisticated models given the ecological services rendered by GS. Given the physiological relationship between extreme heat and CVD, urban heat measured with optical remote sensing archives may not accurately reflect the extreme meteorological situations. One may need to add extreme air-temperature measurements, which would require more sophisticated modelling.

## 4. Conclusions

The results of this study suggest that given the positive effect of green spaces (GSes) harbouring sports facilities in the stimulation of physical activities, GS can advantageously be used to reduce cardiovascular diseases (CVDs) and diabetes. The social services of GS in terms of facilities, particularly those that stimulate physical activities, may be playing a more important role in reducing CVD than in achieving ecological goals and services. Therefore, GS may be considered as an important element in the scope of the global action plan for the prevention and control of non-communicable diseases (NCDs) of the WHO [1]. However, one of the most important conditions is to consider GS impact as defined by their type and proximity. This may imply reconsidering the conceptual framework behind the measurement of accessibility to GS at both domestic and global levels.

## Figures and Tables

**Table 1:** Age standardized and crude rate statistics of the outcomes.

Outcomes	Age Standardized Rates and CI *	Variance	Crude Rates and CI *	Variance
Diabetes	7.70 (7.67–7.73)	0.00	8.78 (8.75–8.81)	0.00
Ischemic heart diseases	7.69 (7.66–7.71)	0.00	9.27 (9.24–9.30)	0.00
Cerebrovascular diseases	2.16 (2.15–2.17)	0.00	2.56 (2.54–2.58)	0.00
Heart failure	3.06 (3.04–3.08)	0.00	3.51 (3.48–3.53)	0.00
Montreal				
Diabetes	8.26 (8.20–8.31)	0.00	9.10 (9.04–9.17)	0.00
Ischemic heart diseases	7.06 (7.01–7.11)	0.00	8.46 (8.40–8.52)	0.00
Cerebrovascular diseases	2.07 (2.04–2.10)	0.00	2.49 (2.46–2.52)	0.00
Heart failure	2.90 (2.86–2.5)	0.00	3.66 (3.61–3.71)	0.00
Quebec				
Diabetes	6.85 (6.76–6.93)	0.00	8.21 (8.11–8.31)	0.00
Ischemic heart diseases	8.31 (8.22–8.40)	0.00	10.58 (10.46–10.69)	0.00
Cerebrovascular diseases	1.79 (1.74–1.83)	0.00	2.26 (2.20–2.31)	0.00
Heart failure	3.38 (3.31–3.46)	0.00	4.13 (4.04–4.22)	0.00

* 99% Confidence intervals.

**Table 2:** Regression model of cerebrovascular prevalence and green spaces with sport facilities for adults ≥20 years living in Montreal and Quebec City CMAs, 2006–2011.

Explanatory Variables	Prevalence Rate Ratio	Standard Error	Z	p > z	95% (Confidence Intervals)
Quartiles of mean distances to GSes with sports facilities					
Q1 (Reference = 0.00–264.59 m)					
Q2 (264.60–468.62 m)	1.03	0.04	0.89	0.37	0.95–1.13
Q3 (468.63–774.42 m)	1.07	0.04	1.69	0.09	0.98–1.17
Q4 (774.43–27,781.92 m)	1.11	0.05	2.26	0.02	1.01–1.22
Social deprivation score *					
Least deprived (reference)					
Intermediate deprivation category	1.04	0.04	1.01	0.31	0.96–1.12
Most deprived	1.03	0.04	0.45	0.45	0.94–1.13
Material deprivation score *					
Least deprived (reference)					
Intermediate deprivation category	0.97	0.03	−0.74	0.46	0.90–1.04
Most deprived	0.96	0.04	−0.81	0.42	0.88–1.05
Quartiles of population density					
Least dense (Reference = 1–2528 km^2^)					
Intermediate 1 (2529–4028 km^2^	0.96	0.04	0.73	0.46	0.88–1.05
Intermediate 2 (4030–8805 km^2^	0.88	0.04	−2.57	0.01	0.80–0.97
Densest (8807–125,463 km^2^)	0.88	0.04	−2.27	0.02	0.80–0.98
Quebec City CMA (in reference to Montreal CMA)	0.86	0.03	−3.25	0.00	0.79–0.94
Proportion of recent immigrants	0.99	0.05	−0.15	0.88	0.98–1.01
Distance to indoor sports centers (in km^2^)	0.99	0.01	−0.35	0.72	0.97–1.01
Distance to major roads (in Km^2^)	1.06	0.06	0.96	0.33	0.93–1.20
Distance to industrial pollutants (in Km^2^)	0.99	0.01	−0.24	0.80	0.96–1.02

Log-Lik Intercept Only: −78,564.381. Log-Lik Full Model: −78,517.428. LR (12): 24.544; Prob > LR: 0.017. * Deprivation index cut-off points: 20% of the cumulative population proportion for the least deprived, 60% for intermediate categories which are grouped into one category in the study and 20% for the most deprived category (rearranged from a quintile, see [24,25]). CMA: Census Metropolitan Areas; GSes: green spaces.

**Table 3:** Regression model of diabetes prevalence and green spaces that have sport facilities for adults ≥20 years living in Montreal and Quebec City CMAs, 2006−2011.

Explanatory Variables	Prevalence Rate Ratio	Standard Error	Z	p > z	95% (Confidence Intervals)
Quartiles of mean distances to GSes with sports facilities					
Q1 (Reference = 0.00–264.59 m)					
Q2 (264.60–468.62 m)	1.04	0.02	1.89	0.06	0.99–1.08
Q3 (468.63–774.42 m)	1.02	0.02	1.35	0.17	0.98–1.07
Q4 (774.43–27,781.92 m)	1.09	0.02	3.47	0.00	1.03–1.13
Social deprivation score *					
Least deprived (reference)					-
Intermediate deprivation category	1.00	0.01	0.13	0.89	0.96–1.03
Most deprived	1.00	0.02	0.15	0.88	0.96–1.04
Material deprivation score *					
Least deprived (reference)					
Intermediate deprivation category	0.99	0.01	−0.42	0.67	0.95–1.02
Most deprived	0.95	0.02	−2.00	0.04	0.91–0.99
Quartiles of population density					
Least dense (Reference = 1–2528 km^2^)					
Intermediate 1 (2529–4028 km^2^	0.95	0.02	−1.97	0.04	0.91–0.99
Intermediate 2 (4030–8805 km^2^)	0.89	0.02	−4.98	0.00	0.85–0.93
Densest (8807–125,463 km^2^)	0.90	0.02	−4.17	0.00	0.86–0.94
Quebec City CMA (in reference to Montreal CMA)	0.95	0.01	−2.42		0.91–0.99
Rate of recent immigrants	0.99	0.00	−1.63	0.10	0.98–1.00
Distance to indoor sports centers (in km^2^)	1.00	0.00	2.37	0.02	0.99–1.01

Log-Likelihood Intercept Only: −78,564.381. Log-Likelihood Full Model: −78,517.428. LR (13): 93.906; Prob > LR: 0.000. * Deprivation index cut-off points: 20% of the cumulative population proportion for the least deprived, 60% for intermediate categories which are grouped into one category in the study and 20% for the most deprived category (rearranged from a quintile, see [24,25]). CMA: Census Metropolitan Areas; GSes: green spaces.

## References

[1] World Health Organization (WHO) (2014). Global Status Report on Non-Communicable Diseases 2014.

[2] Giles-Corti B., Broomhall M., Knuiman M., Collins C., Douglas K., Ng K., Lange A., Donovan R.J. (2005). Increasing walking: How important is distance to, attractiveness, and size of public open space?. Am. J. Prev. Med..

[3] Pretty J., Peacock J., Sellens M., Griffin M. (2005). The mental and physical health outcomes of green exercise. Int. J. Environ. Health Res..

[4] Sugiyama T., Leslie E., Giles-Corti B., Owen N. (2008). Associations of neighbourhood greenness with physical and mental health: Do walking, social coherence and local social interaction explain the relationships?. J. Epidemiol. Community Health.

[5] Lee A.C., Maheswaran R. (2010). The health benefits of urban green spaces: A review of the evidence. J. Public Health.

[6] Alcock I., White M.P., Wheeler B.W., Fleming L.E., Depledge M.H. (2014). Longitudinal effects on mental health of moving to greener and less green urban areas. Environ. Sci. Technol..

[7] Mitchell R., Popham F. (2008). Effect of exposure to natural environment on health inequalities: An observational population study. Lancet.

[8] Richardson E., Pearce J., Mitchell R., Day P., Kingham S. (2010). The association between green space and cause-specific mortality in urban New Zealand: An ecological analysis of green space utility. BMC Public Health.

[9] Tamosiunas A., Grazuleviciene R., Luksiene D., Dedele A., Reklaitiene R., Baceviciene M., Vencloviene J., Bernotiene G., Radisauskas R., Malinauskiene V. (2014). Accessibility and use of urban green spaces, and cardiovascular health: Findings from a Kaunas cohort study. Environ. Health.

[10] Brook R.D., Franklin B., Cascio W., Hong Y., Howard G., Lipsett M., Luepker R., Mittleman M., Samet J., Smith S.C. (2004). Air pollution and cardiovascular disease a statement for healthcare professionals from the Expert Panel on Population and Prevention Science of the American Heart Association. Circulation.

[11] Silverman R.A., Kazuhiko I., Freese J., Kaufman B.J., De Claro D., Braun J., Prezant D.J. (2010). Association of ambient fine particles with out-of-hospital cardiac arrests in New York City. Am. J. Epidemiol..

[12] Cakmak S., Dales R., Leech J., Liu L. (2011). The influence of air pollution on cardiovascular and pulmonary function and exercise capacity: Canadian Health Measures Survey (CHMS). Environ. Res..

[13] Beckerman B.S., Jerrett M., Finkelstein M., Kanaroglou P., Brook J.R., Arain M.A., Chapman K. (2012). The association between chronic exposure to traffic-related air pollution and ischemic heart disease. J. Toxicol. Environ. Health.

[14] Schwartz B.G., Qualls C., Kloner R.A., Laskey W.K. (2015). Relation of total and cardiovascular death rates to climate system, temperature, barometric pressure, and respiratory infection. Am. J. Cardiol..

[15] Cheng C., Campbell M., Li Q., Li G., Auld H., Day N., Pengelly D., Gingrich S., Klaassen J., MacIver D. (2009). Differential and combined impacts of extreme temperatures and air pollution on human mortality in South-Central Canada: Part I: Historical analysis. Air Qual. Atmos. Health.

[16] Coombes E., Jones A.P., Hillsdon M. (2010). The relationship of physical activity and overweight to objectively measured green space accessibility and use. Soc. Sci. Med..

[17] Lim S.S., Vos T., Flaxman A.D., Danaei G., Shibuya K., Adair-Rohani H., Amann M., Anderson H.R., Andrews K.G., Aryee M. (2010). A comparative risk assessment of burden of disease and injury attributable to 67 risk factors and risk factor clusters in 21 regions, 1990–2010: A systematic analysis for the Global Burden of Disease Study 2010. Lancet.

[18] Blais C., Jean S., Sirois C., Rochette L., Plante C., Larocque I., Doucet M., Ruel G., Simard M., Gamache P. (2014). Quebec Integrated Chronic Disease Surveillance System (QICDSS), an innovative approach. Chronic Dis. Inj. Can..

[19] Tu K., Campbell N.R., Chen Z.L., Cauch-Dudek K.J., McAlister F.A. (2007). Accuracy of administrative databases in identifying patients with hypertension. Open Med..

[20] Quan H., Khan N., Hemmelgarn B.R., Tu K., Chen G., Campbell N., Hill M.D., Ghali W.A., McAlister F.A. (2009). Hypertension Outcome and Surveillance Team of the Canadian Hypertension Education Programs. Validation of a case definition to define hypertension using administrative data. Hypertension.

[21] Tu K., Mitiku T., Lee D.S., Guo H., Tu J.V. (2010). Validation of physician billing and hospitalization data to identify patients with ischemic heart disease using data from the Electronic Medical Record Administrative data Linked Database (EMRALD). Can. J. Cardiol..

[22] Tu J.V., Abrahamyan L., Donovan L.R., Boom N. (2013). Best practices for developing cardiovascular quality indicators. Can. J. Cardiol..

[23] Schultz M.G., Otahal P., Cleland V.J., Blizzard L., Marwick T.H., Sharman J.E. (2013). Exercise-induced hypertension, cardiovascular events, and mortality in patients undergoing exercise stress testing: A systematic review and meta-analysis. Am. J. Hypertens.

[24] Statistiques Canada Dissemination Area (DA). http://www12.statcan.gc.ca/census-recensement/2011/ref/dict/geo021-eng.cfm.

[25] Pampalon R., Hamel D., Raymond G. (2004). Indice de Défavorisation Pour L’étude de la Santé et du Bien-Etre au Québec—Mise à Jour 2001.

[26] Desktop Managing Technologies Inc.. http://www.dmtispatial.com/canmap.

[27] Pampalon R., Raymond G.A. (2000). A deprivation index for health and welfare planning in Québec. Chronic Dis. Can..

[28] Escobar J.I. (1998). Immigration and mental health: Why are immigrants better off?. Arch. Gen. Psychiatry.

[29] Ng E. Component of statistics Canada catalogue No. 82-003-X. Health Reports 2011: The Healthy Immigrant Effect and Mortality Rates.

[30] Allender S., Foster C., Hutchinson L., Arambepola C. (2008). Quantification of urbanization in relation to chronic diseases in developing countries: A systematic review. J. Urban Health.

[31] Kheirbek I., Haney J., Douglas S., Ito K., Caputo S., Matte T. (2014). The public health benefits of reducing fine particulate matter through conversion to cleaner heating fuels in New York City. Environ. Sci. Technol..

[32] Environment and Climate Change Canada National Pollutant Release Inventory: Tracking Pollution in Canada. https://www.ec.gc.ca/inrp-npri/Default.asp?lang=En&amp;n=4A577BB9-1.

[33] Zhu Y., Hinds W.C., Kim S., Sioutas C. (2002). Concentration and size distribution of ultrafine particles near a major highway. J. Air Waste Manag. Assoc..

[34] Clougherty J.E., Wright R.J., Baxter L.K., Levy J.I. (2008). Land use regression modeling of intra-urban residential variability in multiple traffic-related air pollutants. Environ. Health.

[35] Janssen N.A.H., Schwartz J., Zanobetti A., Suh H.H. (2002). Air conditioning and source-specific particles as modifiers of the effect of PM (10) on hospital admissions for heart and lung disease. Environ. Health Perspect..

[36] Solecki W.D., Rosenzweig C., Parshall L., Pope G., Clark M., Cox J., Wiencke M. (2005). Mitigation of the heat island effect in urban New Jersey. Glob. Environ. Chang. B Environ. Hazards.

[37] Smargiassi A., Goldberg M.S., Plante C., Fournier M., Baudouin Y., Kosatsky T. (2009). Variation of daily warm season mortality as a function of micro-urban heat islands. J. Epidemiol. Community Health.

[38] Boulfroy E., Khaldoune J., Grenon F., Fournier R., Talbot B. Description de la Méthode Suivie Pour Identifier et Localiser les Îlots de Fraîcheur et de Chaleur. http://www.cerfo.qc.ca/index.php?id=16&amp;no_cache=1&amp;tx_drblob_pi1[downloadUid]=327.

[39] Rao M., George L.A., Rosenstiel T.N., Shandas V., Dinno A. (2014). Assessing the relationship among urban trees, nitrogen dioxide, and respiratory health. Environ. Pollut..

[40] Setälä H., Viippola V., Rantalainen A.L., Pennanen A., Yli-Pelkonen V. (2013). Does urban vegetation mitigate air pollution in northern conditions?. Environ. Pollut..

[41] Sider T., Alam A., Zukari M., Dugum H., Golstein N., Eluru N., Hatzopoulou M. (2013). Land-use and socioeconomics as determinants of traffic emissions and individual exposure to air pollution. J. Transp. Geogr..

[42] Anselin L. (1988). Spatial Econometrics: Methods and Models.

[43] Leyland A.H., Davies C.A. (2005). Empirical Bayes methods for disease mapping. Stat. Methods Med. Res..

[44] Cohen D.A., McKenzie T.L., Sehgal A., Williamson S., Golinelli D., Lurie N. (2007). Contribution of public parks to physical activity. Am. J. Public Health.

[45] Kaczynski A.T., Henderson K.A. (2007). Environmental correlates of physical activity: a review of evidence about parks and recreation. Leisure Sci..

[46] World Health Organization (2003). Urban Planning, Environment and Health: From Evidence to Policy Action. http://www.euro.who.int/__data/assets/pdf_file/0004/114448/E93987.pdf?ua=1.

[47] Barton J., Pretty J. (2010). What is the best dose of nature and green exercise for improving mental health? A multi-study analysis. Environ. Sci. Technol..

[48] Jiang B., Chang C.Y., Sullivan W.C. (2014). A dose of nature: Tree cover, stress reduction, and gender differences. Landsc. Urban Plan..

[49] Jiang B., Li D., Larsen L., Sullivan W.C. (2014). A dose-response curve describing the relationship between urban tree cover density and self-reported stress recovery. Environ. Behav..

[50] Alvarsson J.J., Wiens S., Nilsson M.E. (2010). Stress Recovery during Exposure to Nature Sound and Environmental Noise. Int. J. Environ. Res. Public Health.

[51] Cosley B.J., McCoy S.K., Saslow L.R., Epel E.S. (2010). Is compassion for others stress buffering? Consequences of compassion and social support for physiological reactivity to stress. J. Exp. Soc. Psychol..

[52] Kaczynski A.T., Potwarka L.R., Saelens B.E. (2008). Association of Park Size, Distance, and Features with Physical Activity in Neighborhood Parks. Am. J. Public Health.

[53] Berke E.M., Koepsell T.D., Moudon A.V., Hoskins R.E., Larson E.B. (2007). Association of the built environment with physical activity and obesity in older persons. Am. J. Public Health.

[54] Richardson E.A., Mitchell R., Hartig T., De Vries S., Astell-Burt T., Frumkin H. (2012). Green cities and health: A question of scale?. J. Epidemiol. Community Health.

[55] Samantha H., Ross N.A., Brazeau A.S., Bélisle P., Joseph L., Dasgupta K. (2015). Associations between neighbourhood walkability and daily steps in adults: A systematic review and meta-analysis. BMC Public Health.

[56] Ball K., Timperio A., Simon J., Giles-Corti B., Roberts R., Crawford D. (2007). Personal, social and environmental determinants of educational inequalities in walking: A multilevel study. J. Epidemiol. Community Health.

[57] Gioia A., Paolini L., Malizia A., Oltra-Carrió R., Sobrino J.A. (2014). Size matters: Vegetation patch size and surface temperature relationship in foothills cities of northwestern Argentina. Urban Ecosyst..

[58] Doick K.J., Peace A., Hutchings T.R. (2014). The role of one large greenspace in mitigating London’s nocturnal urban heat island. Sci. Total Environ..

[59] Stahle A. (2010). More green space in a denser city: Critical relations between user experience and urban form. Urban Des. Int..

